# *Bacillus amyloliquefaciens*—Derived Lipopeptide Biosurfactants Inhibit Biofilm Formation and Expression of Biofilm-Related Genes of *Staphylococcus aureus*

**DOI:** 10.3390/antibiotics10101252

**Published:** 2021-10-15

**Authors:** Karolína Englerová, Zdenka Bedlovičová, Radomíra Nemcová, Ján Király, Marián Maďar, Vanda Hajdučková, Eva Styková, Rastislav Mucha, Katarína Reiffová

**Affiliations:** 1 Department of Microbiology and Immunology, University of Veterinary Medicine and Pharmacy, Komenského 73, 041 81 Košice, Slovakia; karolina.englerova@student.uvlf.sk (K.E.); radomira.nemcova@uvlf.sk (R.N.); jan.kiraly@uvlf.sk (J.K.); marian.madar@uvlf.sk (M.M.); vanda.hajduckova@uvlf.sk (V.H.); 2 Department of Chemistry, Biochemistry and Biophysics, University of Veterinary Medicine and Pharmacy, Komenského 73, 041 81 Košice, Slovakia; 3 Equine Clinic, University of Veterinary Medicine and Pharmacy, Komenského 73, 041 81 Košice, Slovakia; eva.stykova@uvlf.sk; 4 Institute of Neurobiology BMC, Slovak Academy of Sciences, Šoltésovej 4-6, 040 01 Košice, Slovakia; mucha@saske.sk; 5 Department of Analytical Chemistry, Institute of Chemistry, Faculty of Science, Pavol Jozef Šafárik University in Košice, Moyzesová 11, 041 54 Košice, Slovakia; katarina.reiffova@upjs.sk

**Keywords:** *Bacillus amyloliquefaciens*, biosurfactant, lipopeptides, *Staphylococcus aureus*, antibiofilm activity

## Abstract

Biosurfactants (BSs) are surface-active compounds produced by diverse microorganisms, including the genus *Bacillus*. These bioactive compounds possess biological activities such as antiadhesive, antimicrobial and antibiofilm effects that can lead to important applications in combating many infections. Based on these findings, we decided to investigate the antibiofilm activity of BSs from the marine *Bacillus amyloliquefaciens* against *Staphylococcus aureus* CCM 4223. Expression of biofilm-related genes was also evaluated using qRT-PCR. Isolated and partially purified BSs were identified and characterized by molecular tools and by UHPLC-DAD and MALDI-TOF/MS. *Bacillus amyloliquefaciens* 3/22, that exhibited surfactant activity evaluated by oil spreading assay, was characterized using the 16S rRNA sequencing method. Screening by PCR detected the presence of the *sfp*, *srfAA, fenD* and *ituD* genes, suggesting production of the lipopeptides (LPs) surfactin, fengycin and iturin. The above findings were further supported by the results of UHPLC-DAD and MALDI-TOF/MS. As quantified by the crystal violet method, the LPs significantly (*p* < 0.001) reduced biofilm formation of *S. aureus* in a dose-dependent manner and decreased expression of biofilm-related genes *fnbA*, *fnbB*, *sortaseA* and *icaADBC* operon. Data from our investigation indicate a promising therapeutic application for LPs isolated from *B. amyloliquefaciens* toward prevention of *S. aureus* biofilm infections.

## 1. Introduction

Antibiotic resistance and nosocomial infections are becoming a major issue in medicines today. Aside from other factors, biofilms contribute to such a situation. Biofilms allow microorganisms to colonize not only tissues and organs but also various medical instruments and equipment and thus significantly contribute to the development and spreading of nosocomial infections. Characteristic features of biofilm-producing microorganisms involve increased resistance against antimicrobials and disinfectants and the ability to resist the immune system of the host. Antibiotic (ATB) therapy of biofilm infections is very demanding and often insufficient and, therefore, these infections may become long-lasting and frequently also regressive [1,2].

*Staphylococcus aureus* is an important opportunistic pathogen with a very variable genome and thus is responsible for a broad spectrum of infections. It belongs to the group of pathogenic microorganisms that are the most frequent agents of communal infections and infections associated with health care [3]. It colonizes mostly the nasal mucosa. These infections are frequently induced by impairment of mucosal barrier and penetration of bacterial cells into the tissues or bloodstream. It is a causative agent of a great number of infections such as acute skin abscesses, bacteraemia, endocarditis and infections of chronic wounds. *S. aureus* infections are complicated and difficult to eradicate, particularly those caused by a methicillin-resistant strain (MRSA) [4]. *S. aureus* belongs among the most common agents of chronic infections associated with biofilm formation. Production of biofilm by these bacteria was most frequently observed on medical implants and host tissues [5]. In the food industry, *S. aureus* biofilm on food contact surfaces poses serious risks of food contamination that cause staphylococcal gastroenteritis in humans [6].

There have been efforts worldwide to look for new approaches or targeted strategies for the discovery of novel antibacterial therapies not associated with the risk of inducing resistance. These include, for example, traditional medicine, enhanced immune stimulation, vaccines, engineered bacteriophages, probiotics, prebiotics and biosurfactants [7,8,9]. Another area of enormous potential is the development of antimicrobial and antibiofilm peptides, also known as host defense peptides (HDPs). These peptides can exert direct antibacterial effects by targeting planktonic cells and exhibit antibiofilm, antiviral, antifungal and host-directed immunomodulatory activities. They are believed to act on multiple targets, thereby possibly lowering the rate of evolution of resistance mechanisms [10,11].

Biosurfactants (BSs) are natural products of several bacterial species defined as surface-active compounds able to reduce the surface and interfacial tension of liquids, solids and gases, allowing these components to be easily mixed or dispersed in the form of emulsions in water or other fluids [12]. BSs are also useful as antibacterial, antifungal, antiviral compounds because they show anti-adhesive activity and cause biofilms to disintegrate [13]. These bioactive compounds possess several advantages when compared to synthetic surfactants, such as low toxicity and irritability, high biodegradability, bioavailability and digestibility, biocompatibility and stability in a wide range of pH, temperature and salinity [14]. Due to these interesting features, they have been extensively used in different fields, such as the food, cosmetics, pharmaceutical or agricultural industries [15]. Rhamnolipids, mannosyl-erythritol lipids and sophorolipids have been used in cosmetic applications in several different formulations, for example, anti-aging skin care products, shower gel, moisturizing skin cleanser, shampoo, and toothpastes [16]. Rhamnolipids and surfactins produced by *Pseudomonas* sp., *Bacillus* sp. and *Acinetobacter* sp. are useful in the removal of polyaromatic hydrocarbons, pentachlorophenol and heavy metals from soil. In addition, rhamnolipids and fengycins have shown potential as biopesticides, fungicides and antizoospore agents [17]. In the food industry, microbial BSs are used in the treatment/cleaning of surfaces coming into contact with food, and as food additives/ingredients. For example, *Saccharomyces cerevisiae* produces a mannoprotein capable of stabilizing water/oil emulsions in biscuits, mayonnaise and ice cream. Lipopeptide bioemulsifier produced by *B. subtilis* significantly improves the texture profile of bread and reduces the sensitivity to microbial proliferation [18]. In the medical field, biosurfactants are not only useful as antibacterial, antifungal, antiviral and anti-adhesive agents (pumilacidin, treahalose lipid, lichenysin, surfactin) but also have a potential for use as major immunomodulatory molecules (mannosylerythritol lipids) and even for anticancer treatment (surfactin, fengycin) and gene therapy (mannosylerythritol lipids) as well as an adjuvant in vaccines (iturin) [19].

Representatives of the genus *Bacillus*, commonly found in marine environments, produce a wide spectrum of antimicrobial and fungicidal compounds [20]. For example, *B. subtilis*, *B. amyloliquefaciens*, and *Bacillus atrophaeus* have the potential to produce secondary metabolites, especially cyclic lipopeptides (LPs) that belong to the family of biosurfactants, which make them useful for agricultural, pharmaceutical and biotechnological applications [21]. These lipopeptide metabolites are divided into three families, surfactins, iturins and fengycins [22], which differ one from another by the length and branching of the fatty acid side chains and by the amino acid substitutions in the peptide ring [23]. Surfactin produced by *B. subtilis* was the first known BS and was isolated by Arima et al. [24]. The exact structure was established by Kakinuma et al. [25]; surfactin is a cyclic LP with a *β*-hydroxy fatty acid as the hydrophobic moiety linked to a specific sequence of seven *α*-amino acids, L-Glu–L-Leu–L-Leu–L-Val–L-Asp–L-Leu–L-Leu, by an amide group and a lactone bond [26]. Iturin is a cyclic peptide of seven amino acids (heptapeptides) linked to a fatty acid (*β*-amino) chain [23], while fengycin is a cyclic decapeptide with a *β*-hydroxy fatty acid in its side chain [27]. In the context of biological control of diseases, these LPs are widely considered as potential alternatives to the growing problem of resistance to conventional antibiotics, fungal infections, and life-threatening diseases [28].

The aim of the study was to isolate BSs from the marine *Bacillus amyloliquefaciens* 3/22 and test their antibiofilm activity against biofilm-producing reference strain *Staphylococcus aureus* CCM 4223 under in vitro conditions. Isolated and partially purified LPs were identified and characterized by molecular tools and by UHPLC-DAD (ultrahigh-performance liquid chromatography with diode array detector) and MALDI-TOF/MS (matrix-assisted laser desorption/ionization time-of-flight/molecular mass). Expression of biofilm-related genes was also evaluated.

## 2. Results

### 2.1. Genotypic Identification of the Isolate

The 16S rRNA sequencing result indicated that the marine isolate belonged to the *Bacillus* genus. BLASTn (basic local alignment search tool nucleotide) analysis (Table 1) showed 100% similarity with *B. amyloliquefaciens*. The sequence was deposited in GenBank and subsequently approved and published with the accession number MN435585.

### 2.2. Presence of Surfactin, Fengycin and Iturin A Genes

PCR (polymerase chain reaction) assays followed by sequencing were conducted to investigate the occurrence of genes for the production of surfactin transcriptional terminator (*sfp*), surfactin synthase subunit 1 (*srfAA*), fengycin synthetase (*fenB*, *fenD*) and malonyl CoA transacylase (*ituD*); the selected *sfp* gene primers amplifieda 675 bp product. Of similar intensity was the detected 201 bp product of amplification of the *srfAA* gene. Selected primers of *fenB* gene failed to amplify the product of size 670 bp. On the contrary, we detected product of the gene *fenD* (269 bp) as well as the gene *ituD* (482 bp). The intensity of amplification products of fragments of genes *fenD* and *ituD* detected in the tested strain was higher in comparison to that of fragments of genes *sfp* and *srfAA*.

BLASTn analysis (Table 1) showed that the *srfAA, sfp* and *ituD* genes were detected in *B. amyloliquefaciens* 3/22 with 97–99% identity. A somewhat lower percentage of identity was observed for the *fenD* gene. The sequence of genes found in this isolate were submitted to GenBank and published under the following accession numbers: MK328493 (*srfAA*), MK328487 (*sfp*), MK328481 (*fenD*), MK328484 (*ituD*).

### 2.3. Ultrahigh-Performance Liquid Chromatography

**Figure 1:**
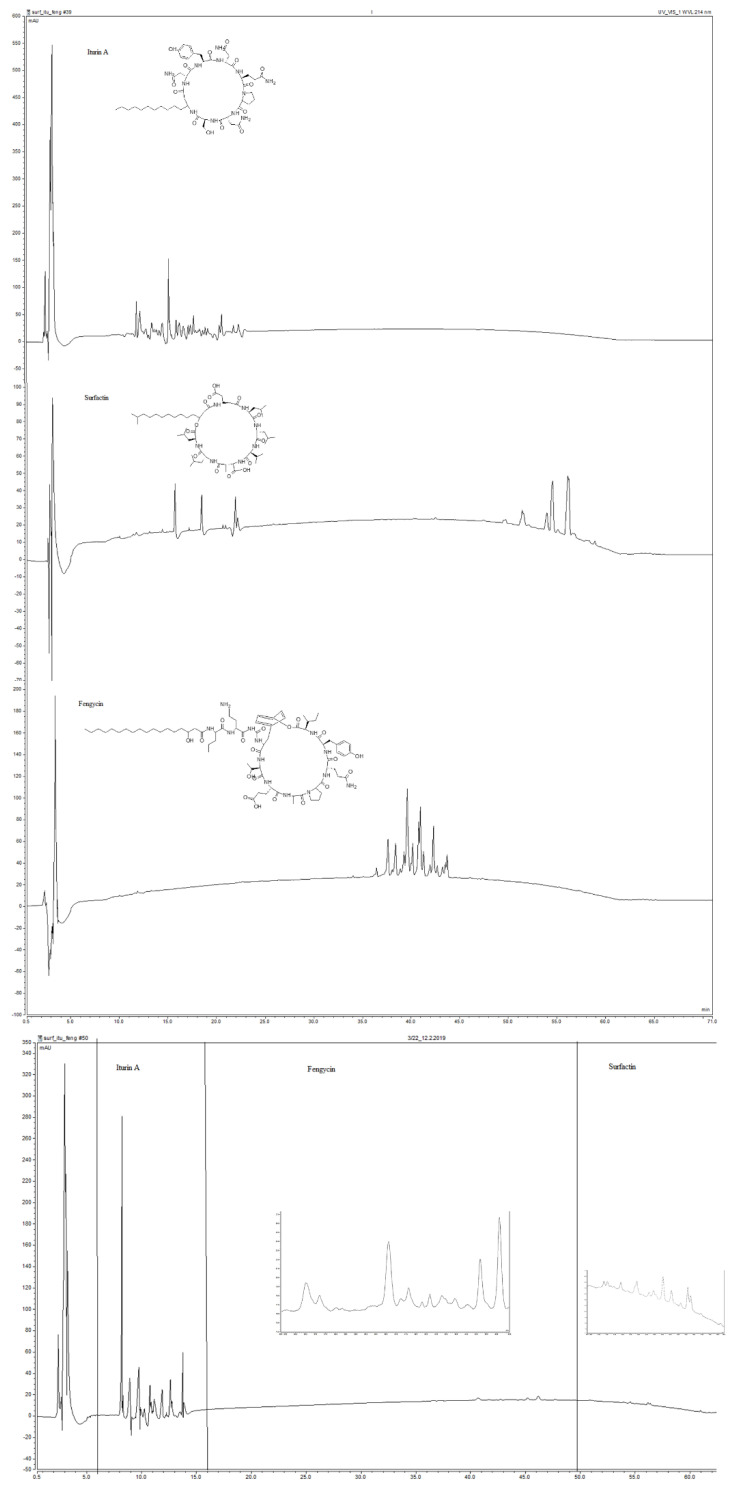
Chromatogram of standards (**up**) and LPs from *B. amyloliquefaciens* 3/22 (**down**), containing components of iturin A, surfactin and fengycin.

The UHPLC method with DAD detector was used for the immediate detection of studied lipopeptides. The chromatograms of standards—fengycin, iturin A, and surfactin are presented in Figure 1 (up). The flow rate, mobile phases ratio and injection volumes were varied to find out the method for good separation of studied matrices. The mobile phase consisted of 0.025% trifluoroacetic acid (TFA) in acetonitrile (eluent A) and water (eluent B). As the chromatograms of the mentioned standard lipopeptides show, we observed three main groups of peaks. The iturin A standard retention time was detected in the range of 11.25–22.5 min. Then we attributed the peaks for fengycin standard at 35.0–45.0 min. Finally, the three surfactin standard peaks were eluted at 15.73, 18.46 and 20.90 min, connected with a broad group of peaks in the scale of 51.0–60.0 min [29]. As can be seen in Figure 1 (down), the UHPLC chromatogram of the studied isolates of *B. amyloliquefaciens* 3/22 LPs contained the peaks of mentioned lipopeptides isomers attributed by comparing the retention times. The retention times for iturin A isomers presented in the studied isolate of *B. amyloliquefaciens* 3/22, were 11.77, 12.38, 13.13, 14.39, 15.01, 15.89, 16.13, and 16.68 min. The retention times for surfactin isomers were observed at 15.61, 18.50, 22.19, 51.44, 53.94, 54.54, 54.64, 55.90, 56.10, and 56.26 min. The fengycin isomers were eluted at 36.40, 37.23, 38.34, 39.28, 40.11, 40.71, 41.04, and 42.30 min. This confirmed the results of the genetic analysis mentioned in this article.

### 2.4. MALDI-TOF/MS Analysis

**Figure 2:**
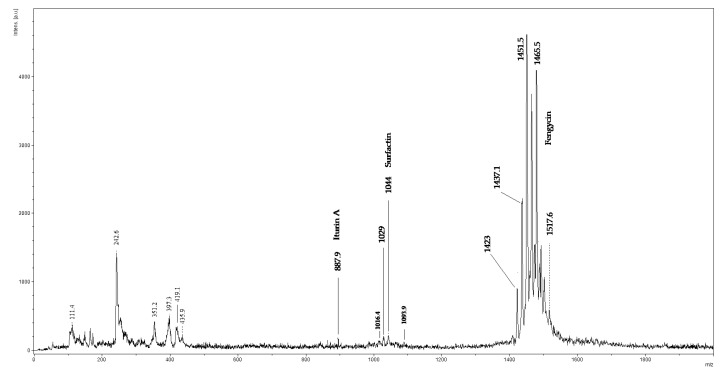
MALDI-TOF/MS Spectra of LPs Isolated from *B. amyloliquefaciens* 3/22.

The lyophilized extract of LPs from *B. amyloliquefaciens* 3/22 was subjected to MALDI-TOF/MS analysis as an acetonitrile solution. In the spectra of isolated LP, peaks with masses very similar to LP compounds were detected. As shown in Figure 2, the most intensive signals in the *m*/*z* range of 1400–1600 were observed in the MALDI-TOF/MS spectra of the isolated lipopeptide mixture. The peak at *m*/*z* = 887.9 was attributed to iturin A, the peaks at *m*/*z* = 1029 and 1044 were attributed to surfactin and the ions between *m*/*z* = 1400–1600 were regarded as belonging to fengycin (1479.7, 1465.5, 1451.5, 1437.1, 1423). Results obtained by MALDI-TOF/MS and HPLC methods confirmed that *B. amyloliquefaciens* 3/22 produces surfactin, fengycin and iturin A, the three families of lipopeptide biosurfactants.

### 2.5. The Effect of LPs 3/22 on S. aureus CCM 4223 Biofilm Formation

**Figure 3:**
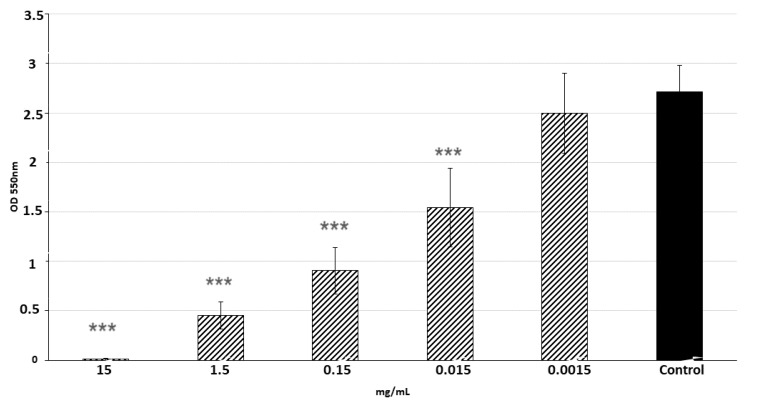
The effect of LPs 3/22 on *S. aureus* CCM 4223 biofilm formation. Control—*S. aureus* CCM 4223 in mBHI broth (brain heart infusion) without LPs 3/22; data are presented as the means ± standard deviation; *** (*p* < 0.001)—significant difference compared to the non-treated control.

Antimicrobial activity of LPs 3/22 against *S. aureus* in planktonic cells was determined by MIC (minimal inhibitory concentration). The results showed that MIC value was 15 mg/mL. Inhibition of biofilm formation by LPs 3/22 was compared with the control by analyzing absorbance of crystal violet. As shown in Figure 3, the LPs produced by *B. amyloliquefaciens* 3/22 significantly (*p* < 0.001) reduced biofilm formation by *S. aureus* CCM 4223 in a dose-dependent manner. The biofilm formation by the indicator strain was completely inhibited when concentration of LPs 3/22 reached 15 mg/mL. Concentrations of LPs 3/22 equal to 1.5 and 0.15 mg/mL inhibited the formation of biofilm by more than 50%. The percentage of inhibition of biofilm formation lower than 50% was observed at concentrations of LPs 0.015 mg/mL (Table 2).

### 2.6. Analysis of qRT-PCR Results

**Figure 4:**
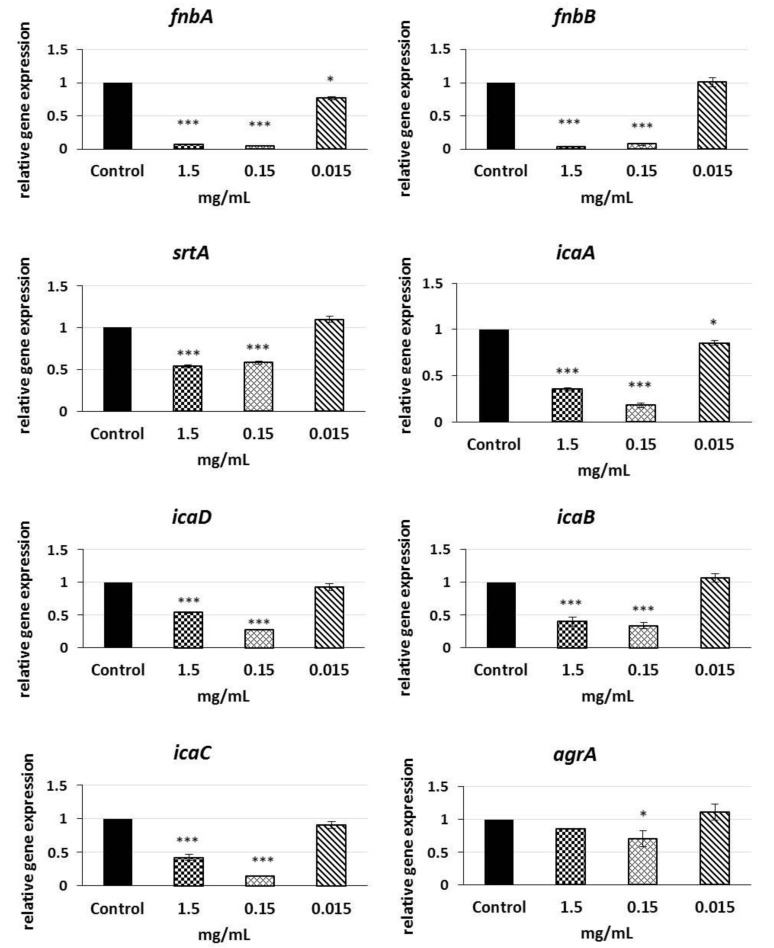
The effect of LPs 3/22 on mRNA (messenger RNA) decreased expression of biofilm-related genes in *S. aureus* CCM 4223; data are presented as means ± standard deviation; * (*p* < 0.05) and *** (*p* < 0.001)—significant difference compared to the non-treated control.

We conducted qRT-PCR (quantitative real-time polymerase chain reaction) using primers described elsewhere to determine whether expression of biofilm-related genes in *S. aureus* biofilms was regulated by subinhibitory (1.5; 0.15; 0.015 mg/mL) concentrations of LPs 3/22. Figure 4 shows that LPs produced by *B. amyloliquefaciens* 3/22 modulated the expression of biofilm-related genes in *S. aureus* CCM 4223. The results of measuring the expression level of *fnbA* and *fnbB* genes indicated a significant 14.37- and 29.56-fold down-regulation when the culture media were supplemented with 1.5 mg/mL of LPs 3/22 and 22.94- and 13.67-fold down-regulation when they were supplemented with 0.15 mg/mL of LPs 3/22. The expression level of *fnbA* gene was significantly decreased (1.3-fold) also at the LPs 3/22 concentration of 0.015 mg/mL. Transcription levels of *sortaseA* gene in biofilms that were treated with 1.5 mg/mL and 0.15 mg/mL of LPs 3/22 decreased by 1.86- and 1.72-fold, respectively, compared with the non-treated control. As observed from the results, *icaA* gene was significantly down-regulated by 2.82-, 5.5- and 1.17-fold when the culture media were supplemented with 1.5 mg/mL, 0.15 mg/mL and 0.015 mg/mL of LPs 3/22, respectively. Similarly, at the LPs 3/22 concentration of 1.5 mg/mL the genes *icaD*, *icaB* and *icaC* were inhibited by 1.85-, 2.25- and 2.37-fold, respectively, and at the LPs 3/22 concentration of 1.5 mg/mL by 3.72- 2.98- and 6.99-fold, respectively. The expression level of *agrA* gene was significantly decreased by 1.42-fold at the LPs 3/22 concentration of 0.15 mg/mL compared with the non-treated control.

## 3. Discussion

In the present study, BSs produced by marine strain *B. amyloliquefaciens* 3/22 were characterized and evaluated for their antibiofilm activity in vitro. PCR analysis revealed that this strain possessed the genes coding for the simultaneous co-production of 3 LPs: surfactin (*sfp*, *srfAA*), fengycin (*fenD*) and iturin (*ituD*). Several authors also detected the presence of genes encoding the co-production of three or more LPs by *Bacillus* spp. Plaza et al. [30] demonstrated the co-production of iturin (*ituC, ituD*), fengycin (*fenB*, *fenD* and surfactin (*srfAA*) by three strains of *Bacillus subtilis* KP7, T’-1 and I’-1a. Zhang et al. [31] found LP genes (*fenB*, *sfp*, and *ituD*) in *B. amyloliquefaciens* W10 isolated from tomato rhizosphere. Xu et al. [32] first identified cyclic lipopeptides from *Bacillus siamensis*. The results of these authors showed that PCR products of *sfp*, *srfD*, *fenB*, *ituA*, and *ituC* were amplified. He et al. [33] reported the presence of the LP genes *sfp* (surfactin), *fenB* (fengycin), *ituD* (iturin) in *B. subtilis* Czk1, which was obtained from the aerial roots of rubber trees. It was contemplated that the co-production of several LPs by one strain can increase their synergistic effect [34]. Iturin A and fengycin separately exhibited antifungal activity. Surfactin exhibits antibacterial properties and acts in a synergistic mode through enhancing the antifungal activity of iturin A [35]. Genes responsible for the production of LPs were detected also in other representatives of the genus *Bacillus*. The strain *B. subtilis* Bbv 57 was positive for iturin (*ituD* gene) and surfactin (*srfA* gene; *sfp* gene) lipopeptides [36]. Co-production of iturin and surfactin by additional *Bacillus* spp. was also described. The strains *Bacillus cereus* UASBR3, *B. cereus* UASBR6, *B. subtilis* UASBR5, *B. pumilus* UASBR8, and *B. amyloliquefaciens* UASBR9 have been known to possess the *srfAA* and *ituC* genes [37]. The presence of genes encoding *ituD* and *sfp* were detected in *Bacillus* sp. P5 isolated from puba (food made by spontaneous fermentation of cassava roots), suggesting the production of iturin A and surfactin by this strain [38]. In another study, the presence of the *srfAA* gene was detected in 15 strains of *B. subtilis*, *B. pumilus*, *Bacillus megaterium* and *B. amyloliquefaciens* isolated from potato rhizosphere in Iran. In addition, the *fenD* gene was detected in 80% and *ituC* in 66.7% of the tested strains [39]. Co-production of fengycin (*fenB*) and iturin (*ituA*) was detected in *B. subtilis* YB-05 [40]. Similarly, PCR detection showed that *B. amyloliquefaciens* PG12 isolated from apple fruit possessed the genes *ituD, ituC, fenB, fenC* and *fenF*, which are responsible for the production of fengycin and iturin [41].

Lipopeptides isolated from *B. amyloliquefaciens* 3/22 strain were identified by UHPLC-DAD and MALDI-TOF/MS analysis. The LC methods are standardly used for the biosurfactants characterization [41,42,43]. The example of a three-stage strategy using the gradient elution was published by Yang [40]. As eluent authors used the acetonitrile-water mixture in various ratios at the flow rate of 0.8 mL/min. The analysis time was 25 min. Under the mentioned conditions, our separation process of LPs failed, so we changed the eluent by adding trifluoroacetic acid (TFA) [43]. Better separation was observed by the addition of 0.025% TFA into acetonitrile and water. Finally, we identified iturin A, fengycin and surfactin LPs at 11–17 min, 36–43 min, and 51–57 min, respectively. In summary, we can confirm that under the changed conditions of elution, we were able to separate the studied lipopeptide biosurfactants of 3/22. The MALDI-TOF/MS analysis is a broadly used methodology for lipopeptides identification. This method is sensitive and reveals the ion sequences of cyclic peptides [40]. For the 3/22 LPs identification, we noticed ions at *m*/*z* = 1093.9 (iturin A), 1016.4, 1029 and 1044 (surfactin) and 1465.5 (fengycin). These observations are in good agreement with the literature, for example, the study of the *B. subtilis* CMB32 strain by Kim et al. [44], in which the authors identified peaks in the range of 1016 and 1044 for surfactin, fengycin was observed at *m*/*z* 1452 and 1542, and between 1066 and 1094 iturin peaks were detected. A comparable mass intensity was found in another study by Bernat et al. [45].

BSs are very attractive natural molecules with antimicrobial and antibiofilm properties due to their amphiphilic nature [46]. The antimicrobial action of BSs involves their ability to disrupt membrane integrity, leading to cell lysis and metabolite leakage. Moreover, changes in the membrane structure and impairment of proteins conformations alter the essential membrane functions including production and transport of energy [47,48]. In addition to antimicrobial action, the antibiofilm activity of BSs is associated also with their ability to affect adhesion and dislodgement of bacteria from the surface due to the changes in surface tension and bacterial cell-wall charge [49] and their influence on expression of biofilm-related genes [50,51].

Despite the advances made in studying the *S. aureus* biofilm formation, its architecture and role in pathogenesis and drug resistance [52], less is known about the mechanism of action of LPs in inhibiting this biofilm. The results of experiments carried out during co-incubation (effect on biofilm formation and pre-formed biofilms) or pre-coating (preventing microbial adhesion) indicate the antibiofilm action of LPs against potential pathogens including *S. aureus*.

Giri et al. [53] evaluated the anti-adhesive activities of the LPs from *B. subtilis* VSG4 and *B. licheniformis* VS16 against *S. aureus* ATCC 29523, *Salmonella typhimurium* ATCC 19430, and *Bacillus cereus* ATCC 11778. The pre-coating assays showed that the LPs exhibited anti-adhesive activity, even at concentrations of 3–5 mg/mL and caused the biofilm eradication with percentages ranging from 63.9 to 80.03% for VSG4 biosurfactant, and from 61.1 to 68.4% for VS16 biosurfactant. Janek et al. [54] reported the ability of the cyclic lipopeptide pseudofactin II (0.5 mg/mL) to prevent formation of biofilm by *Escherichia coli*, *Enterococcus faecalis*, *Enterococcus hirae*, *Staphylococcus epidermidis*, *Proteus mirabilis* and *Candida albicans* on a polystyrene surface. De Araujo et al. [55] observed that surfactin at 0.50% (*w*/*v*) significantly reduced adhesion of *Listeria monocytogenes* to a polystyrene surface and at higher concentrations achieved as high as 54% inhibition. In another study by Abdelli et al. [47] it was observed that surfactin obtained by *Bacillus safensis* F4, at concentrations of 5 and 10 mg/mL, exhibited anti-adhesive activity against the biofilm forming by *S. epidermidis* S61, which exceeded 80%. Rivardo et al. [56] demonstrated specific anti-adhesion activity of two biosurfactants from *B. subtilis* and *B. licheniformis*. The V9T14 biosurfactant inhibiting biofilm adhesion of *E. coli* CFT073 was ineffective against the *S. aureus* ATCC 29213 biofilm and, on the contrary, the V19T21 biosurfactant inhibited adhesion of *S. aureus* ATCC 29213 but was ineffective against *E. coli* CFT073. Biofilm formation by *E. coli* and *S. aureus* was decreased by 97% and 90%, respectively. The antiadhesive activity against biofilm of both strains was attributed to the fraction belonging to the fengycin-like family obtained by flash chromatography from each purified biosurfactant. Cordeiro et al. [57] observed that the co-incubation of the *B. subtilis* biosurfactant TIM96 (mixture of surfactin, iturin and fengycin) with clinical strains of *Trichosporon* reduced adhesion of fungal cells by up to 96.89% and caused up to a 99.2% reduction in the metabolic activity of mature *Trichosporon* biofilms, decreasing their thickness and cell viability. A complex of surfactants PPE (polymyxin D1, fusaricidin B and traces of surfactin) isolated from *Paenibacillus polymyxa* at a concentration of 2 mg/mL, inhibited (87–98%) formation of many Gram positive bacterial biofilms including those produced by *S. aureus* [58]. Surfactin isolated from *B. amyloliquefaciens* NS6 exhibited dispersion activity against the biofilm of *Streptococcus mutans*, when, at the highest concentration of 80 mg/mL the dispersion reached 62% [59]. Meena et al. [60] reported that biofilms of pathogenic bacterial strains *S. aureus* ATCC 6538, *Pseudomonas* sp., *Klebsiella pneumoniae, E. coli* NCTC 10418, *Salmonella typhi* and *S. typhimurium* NCTC74, treated with surfactin isolated from *B. subtilis* KLP2015 (100 µg/mL), were reduced by 58.10%, 47.86%, 14.83%, 13.91%, 11.01% and 10.23%, respectively.

Results of the present study showed that the co-incubation of the mixture of surfactin, fengycin and iturin from *B. amyloliquefaciens* 3/22 with *S. aureus* CCM 4223 significantly inhibited biofilm formation in a dose-dependent manner. The percentage of inhibition of biofilm formation at concentrations of 15, 1.5, 0.15 and 0.015 mg/mL ranged from 100 to 39%. Antimicrobial activity of LPs 3/22 has been tested preliminary in an antimicrobial assay. MIC value was 15 mg/mL. This means that the concentrations 1.5, 0.15 and 0.015 mg/mL had no inhibitory effect on the growth of the indicator strain. Our previous investigations under the pre-coating conditions showed that LPs 3/22 significantly affected *S. aureus* CCM 4223 adhesion to a polystyrene microplate (*p* < 0.01; *p* < 0.05) and strongly promoted the biofilm dislodging [61].

Another part of our study was devoted to the analysis of expression of biofilm-related *fnbA*, *fnbB*, *srtA*, *icaADBC* and *agrA* genes by means of qRT-PCR in order to investigate the potential mechanisms that could form the basis of the observed reduced growth of biofilm in the presence of LPs 3/22. All the investigated genes were involved in the regulation of biofilm formation by *S. aureus*. To the best of our knowledge, no reports are available on the abilities of *B. amyloliquefaciens* LPs as regulators of *S. aureus* biofilm-related genes.

The sortaseA enzyme is responsible for covalent anchoring of surface adhesive proteins in the cell wall biosynthesis and thus the excessive expression of the *srtA* gene contributes to the virulence of *S. aureus* [62]. The most important adhesins that facilitate attachment of bacterial cells to the biotic surface during the first stage of adhesion include fibronectin binding proteins A and B (FnBPA, FnBPB) [63]. Due to their important influence on the virulence of bacteria, the sortaseA transpeptidase and fibronectin binding proteins constitute a potential target of the development of vaccines and therapeutic strategies [64,65]. Our study showed that LPs 3/22 exhibiting a strong negative influence on biofilm formation, significantly (*p* < 0.001) reduced expression of genes *fnbA* and *fnbB* and, at the same time, decreased expression of the gene encoding enzyme sortaseA. It has been established that inhibition of the enzyme sortaseA and fibronectin binding proteins brings about impaired development of biofilm which is manifested by its reduced accumulation [66,67].

*S. aureus* produces polysaccharides of intercellular adhesion (PIA), the release of which is controlled by operon *icaADBC*. *Ica* operon participates in regulation of the release of autoinducer-2 signaling molecules in *S. aureus*, by means of which bacteria can sense population density (*quorum sensing*). These molecules play an important role in intraspecies and interspecies communication [68]. Inhibition of PIA production results in biofilm reduction mediated by genetic regulation of *ica* operon genes [69,70]. The accessory gene regulator (*agr*) is also associated with *quorum sensing*. The *agrB* and *agrD* genes regulate expression and transport of the autoinducing peptide. As soon as a sufficient amount of the autoinducing peptide aggregates in the surrounding extracellular environment, a two-component system *agrA* and *agrD* triggers intracellular communication and sensing of the population density [71]. Our results showed that LPs 3/22 at sub-inhibition concentrations significantly down-regulated (*p* < 0.001) the *icaADBC* genes expression. *AgrA* gene expression level was significantly affected (*p* < 0.05) when the concentration of LPs 3/22 reached 0.15 mg/mL. Similar significant down-regulation of expression of *icaA* and *icaD,* as well as alteration of the *quorum sensing* system by the regulation of the auto inducer 2 was observed by Liu et al. [72] after treatment of *S. aureus* biofilm with surfactin from *B. subtilis*. Cramton et al. [73] observed that deletion of *ica* locus significantly decreased formation of biofilm by *S. aureus*.

## 4. Materials and Methods

### 4.1. Microorganisms

The *Bacillus amyloliquefaciens* 3/22 strain was isolated from the seaweed sample of the Adriatic Sea and identified using the 16S rRNA sequencing method as described below. The indicator strain *Staphylococcus aureus* CCM 4223 was obtained from the Czech Collection of Microorganisms (Brno, Czech Republic). *S. aureus* was cultivated in brain heart infusion broth (HiMedia Laboratories, Mumbai, India) with 1% glucose and 2% NaCl (mBHI broth) for biofilm production. The reference surfactin-producing strain *Bacillus subtilis* subsp. *subtilis* DSM 3257 was obtained from Leibniz Institute DSMZ-German Collection of Microorganisms and Cell Cultures.

### 4.2. Isolation and Screening of Isolates for BSs Production

A 0.5 g seaweed sample was homogenized (Stomacher Lab Blender 80, Seward Medical Limited, London, UK) with 4.5 mL of a sterile diluent. A series of 10-fold dilutions was prepared in an isotonic saline solution. From the appropriate dilutions, 0.1 mL aliquots were spread onto brain heart infusion agar (BHI agar pH 7; HiMedia) and the plates were incubated at 27 °C for 48 h under aerobic conditions. Using the Gram and Wirtz-Conklin staining methods, preparations were made from the pure colonies, and the microscopic images—shape, color, size, arrangement and the presence of spores—were observed and evaluated. Gram-positive, spore-forming, rod-shaped isolates were tested for BSs production. BHI broth (HiMedia) was used as a seed medium, which was inoculated with a loop-full of the previously obtained isolates on BHI agar and incubated at 27 °C for 18 h. Subsequently, a liquid McKeen medium [74] (20 g/L glucose, 5 g/L glutamic acid, 1 g/L K_2_HPO_4_, 1.02 g/L MgSO_4_, 0.5 g/L KCl, pH 7.0) supplemented with 1 mL of mineral solution (0.5 g/L MnSO_4_·7H_2_O, 0.16 g/L CuSO_4_·5H_2_O, 0.015 g/L FeSO_4_·7H_2_O) was inoculated with 2% (*v*/*v*) seed media. After inoculation, the flasks were incubated on a water bath shaker (JULABO SW 2C, Labor Technic GMBH Selbach, Germany) at 27 °C and 120 rpm for 72 h. The cell-free supernatants (CFS) obtained by centrifugation (4754× *g*/45 min/4 °C) were screened for BSs production by an oil spreading test. This test was performed according to Morikawa et al. [75] with the following modification: 20 µL of crude oil (Slovnaft, Vlčie hrdlo, Slovakia) was added to the surface of 10 mL of distilled water in a 60 mm diameter Petri dish to form a thin oil layer. Then, 100 µL of CFS was gently applied to the centre of the oil layer. In a positive case, the oil was displaced, and a clearing zone was formed. The diameter of this clearing zone on the oil surface correlated with the surfactant activity (oil displacement). The reference surfactin-producing strain *B. subtilis* subsp. *subtilis* DSM 3257 was used as a positive control, and McKeen medium served as a negative control. The 3/22 isolate that produced the largest clearing zone was selected for further study.

### 4.3. Genotypic Identification

DNAzol Direct (Molecular Research Center Inc., Cincinnati, OH, USA) was used for DNA isolation according to the manufacturer’s instructions. A 1 µL volume of DNA sample was added to One Taq 2× Master Mix (New England BioLabs, Ipswich, MA, USA) in a total volume of 50 µL and amplified by the PCR method to detect 16S ribosomal RNA (rRNA) genes using the following universal primers: Bac27F (5-AGAGTTTGATCMTGGCTCAG-3) and 1492R (5-CGGYTACCTTGTTACGACTT-3) synthesized by Merck–Sigma Aldrich company (Darmstadt, Germany). The expected size of the PCR fragment was 1465 bp [76]. The amplification programme consisted of the following cycle conditions: initial activation for 5 min at 94 °C; 31 cycles of 1 min at 94 °C; annealing for 1 min at 55 °C; extension step of 3 min at 72 °C; final 10 min extension step at 72 °C. The PCR was performed on a thermocycler (TProfessional Basic, Biometra GmbH, Göttingen, Germany). A 10 µL aliquot of the product was mixed with 2 µL of the mixture composed of 1667 µL of 6× DNA loading buffer (Thermo Fisher Scientific, catalogue number: R0611) and 2 µL of 10,000× GelRed™dye (Biotium Inc., Hayward, CA, USA). The PCR product was then separated by horizontal 1% agarose gel electrophoresis in Tris-borate-EDTA buffer (pH 7.8) and visualized under UV light. The amplification product was purified, and DNA sequencing was performed with Microsynth AG (Balgach, Switzerland). The obtained forward and reverse reads were validated and assembled using *Geneious 4.8.5.* Software (Biomatters, San Diego, CA, USA). The species was identified based on the consensus sequence of the 16S rRNA gene and by genotyping using online BLASTn analysis (https://BLAST.ncbi.nlm.nih.gov/BLAST.cgi, accessed on 16 September 2019). The validated sequence was sent to GenBank, and the accession number of *B. amyloliquefaciens* 3/22 was obtained.

### 4.4. Detection of the LP Genes

Genomic DNA from *Bacillus amyloliquefaciens* 3/22 was isolated using a High Pure PCR Template Preparation Kit (ROCHE, Indianopolis, IN, USA). PCR amplifications were carried out in 20 µL reaction mixtures containing 7 µL of nuclease-free water, 10 µL of Thermo Scientific DreamTaq Green PCR Master Mix (2×), 1 µL of each 10 µM primer and 1 µL of bacterial DNA (10 ng/µL) isolated by the kit. PCR amplifications were performed using a GenePro (BIOER, Hangzhou, China) thermocycler. The following parameters were used: initial activation at 95 °C for 3 min; 35 cycles of denaturation at 94 °C for 1 min; annealing for 30 s at variable temperatures depending on the primers used; and an extension step at 70 °C for different times (due to the size of the primers used). Finally, the amplification was completed by the extension step at 70 °C for 10 min. The reference surfactin-producing strain *B. subtilis* subsp. *subtilis* DSM 3257 was used as a positive control. The experiment included a negative control mixture without added DNA. The final product of the amplification reaction was analyzed by electrophoresis using a 1.5% agarose gel with 2 µL GoodView^TM^ (Ecoli) and evaluated by UV light visualization. The primers of genes used in the PCR amplification were selected based on the published data (Table 3). The products obtained from the PCRs were purified and sequenced using primers from both directions (Microsynth AG Postfach 58 6961 Wolfurt-Bahnhof Austria). The final sequences were compared with other bacterial sequences of the genes coding for biosynthesis of BSs in the National Center for Biotechnology Information (NCBI) database using BLASTn analysis. The software Sequin (https://www.ncbi.nlm.nih.gov/Sequin/, accessed on 27 July 2019) was used for sending the sequences of BSs to the GenBank database. The GenBank accession numbers of BSs genes were obtained.

### 4.5. Isolation of Biosurfactants

BSs were extracted using the method by Plaza et al. [30] with slight modifications. Briefly, 300 mL aliquot of McKeen medium was inoculated with 3% (*v*/*v*) *Bacillus amyloliquefaciens* 3/22 and incubated in a rotary shaker (Shaker SKO-D XL, ARGOlab, Carpi, Italy) at 27 °C with shaking (140 rpm) for 72 h. The bacterial culture was made cell free by centrifugation at 4800× *g* for 65 min; the collected filtrate was acidified by 6 M HCl to pH 2. The precipitate that formed overnight at 4 °C was centrifuged again. The sediment was dissolved in 100 mL of distilled water and the pH of the solution was adjusted by 1 M NaOH to a value of 7. The LPs were extracted with ethyl acetate and methanol at a ratio of 4:1 (*v*/*v*). After combination, the organic layers were dried by sodium sulphate. The filtered solvent was concentrated in a rotary evaporator (IKA RV 10 Digital, IKA Germany) and the LPs (yellow oily product) were lyophilized and then stored at −70 °C.

### 4.6. Ultrahigh-Performance Liquid Chromatography

The extracted and purified mixtures of lipopeptides were dissolved in acetonitrile and then filtered using a 0.22 μm membrane filter. For analyses, the UHPLC Dionex UltiMate 3000 system with DAD detector at a wavelength of 214 nm was used. For separations, the reverse-phase column YMC Meteoric Core C18 Bio (150 × 4.6 mm) with a particle size of 2.7 μm was utilized. The injection volumes were 20 μL. All the standard lipopeptides (iturin A, fengycin, surfactin) were purchased from Sigma Aldrich, Germany. The concentration of standards was 0.5 mg/mL. The eluent A consisted of 0.025% TFA in acetonitrile and eluent B was 0.025% water solution of TFA. The separation strategy was optimalized using gradient elution and at the 0–5 min mark 100% of eluent B was used with the flow rate of 0.5 mL/min; the linear gradient followed from 5 to 10 min, using 0–10% of eluent A with the flow rate of 0.8 mL/min, and finally, from 10 to 75 min, 10–90% of eluent A with the flow rate of 1.5 mL/min was performed.

### 4.7. MALDI-TOF/MS Analysis

Studied LPs were analyzed using matrix-assisted laser desorption/ionization time-of-flight (MALDI-ToF MS Biotyper, Bruker Daltonics, Germany) to determine molecular mass. An amount of 1 μL of the sample was inserted to a target plate (Bruker Daltonics, Germany, MSP Target) and dried at room temperature. 2-α-cyano-4-hydroxycinnamic acid, HCCA (Sigma, Saint Louis, MO, USA) was used as a matrix. For desorption and ionization, the UV laser at the wavelength of 337 nm at 20 kV voltage was used. The mass spectra were obtained using the mass spectrometer Microflex LT including flexControl 3.0 software (Bruker Daltonics, Bremen, Germany). The values of *m*/*z* were measured randomly by 100 laser shots in the range of 800–2000. FlexAnalysis 3.0 software (Bruker Daltonics, Bremen, Germany) was used for analyses. The peaks detection was performed using a Centroid detection algorithm with a signal-to-noise threshold of 1, a relative intensity threshold of 0%, a minimum threshold of 0, and a peak width of 0.2 *m*/*z* [79].

### 4.8. Determination of Growth Inhibition Activity of LPs 3/22 against S. aureus in Planktonic Cells

MIC of LPs 3/22 against *S. aureus* CCM 4223 was determined using broth micro-dilution assay. Serial twofold dilutions of LPs 3/22 resulting in concentrations ranging from 0.058 mg/mL to 30 mg/mL were prepared in BHI broth in a 96-well plate (100 μL per well). A diluted bacterial suspension was added to each well to control the final concentration of 1 × 10^6^ CFU/mL. The wells with media only were used as negative controls. By contrast, wells containing no LPs 3/22 in media, but only inoculated bacteria, were used as positive controls. The plates were incubated for 24 h at 37 °C and the lowest concentration with any visible bacterial growth were considered the MIC. The assay was performed in triplicate.

### 4.9. Effect of LPs on Biofilm Formation

A modified microplate assay version of the previously described method of O’Toole et al. [80] was used for assaying the biofilm formation. Concentrations of LPs from *B. amyloliquefaciens* 3/22 that ranged from 15 mg/mL to 0.0015 mg/mL (tenfold dilution) were obtained in 96-well plates with 100 μL of mBHI broth per well (Greiner ELISA 8 Well Strips, 350 μL, Flat Bottom, Medium Binding; Cruinn Diagnostics Ltd., Dublin, Ireland). Afterward, bacterial suspension (100 μL) of *S. aureus* CCM 4223 (McFarland 0.5) was inoculated, and the plates were incubated for 24 h at 37 °C. We used mBHI with saline or mBHI with relevant concentrations of LPs 3/22 as negative controls. The mBHI broth with an indicator strain without LPs 3/22 served as a positive control. After incubation, the supernatant was aspirated from the wells. The biofilm formed in the well of the microtitre plate was gently washed 3 times with 200 µL of phosphate-buffered saline (PBS, pH 7.4; containing 8 g/L NaCl, 0.0002 g/L KCl, 1.15 g/L Na_2_HPO_4_, 0.2 g/L KH_2_PO_4_) and dried at 25 °C for 40 min. The remaining attached bacteria were stained for 30 min at 25 °C with 200 µL of 0.1% (*m*/*v*) crystal violet in an isopropanol-methanol-PBS solution (1:1:18 *v*/*v*). The dye solution was aspirated away, and the well was gently washed 3 times with 200 mL of distilled water. After the water was removed and the cells were dried for 30 min at 25 °C, the dye bound to the adhered biofilm was extracted with 200 µL of 33% (*v*/*v*) glacial acetic acid in distilled water. A 150 µL aliquot was transferred from each well to another microplate for determination of optical density (OD) at 550 nm using a Synergy 4 Multi-Mode Microplate Reader (BioTek, Winooski, VT, USA). The experiment was conducted in triplicate and the results are presented as the means ± standard deviation (SD). Percentage inhibition of biofilm formation was calculated according to the formula (1) as described in the study of Jadhav et al. [81]. ABSs represent the absorbance of the well with the test strain and BSs and Ao absorbance of the well with the test strain without BSs.

Percentage inhibition = [1 − (ABSs/Ao)] × 100(1)

### 4.10. Quantification of Gene Expression Using qRT-PCR

LPs diluted in mBHI medium were inoculated with 0.5 McFarland *S. aureus* CCM4223 into the concentrations of 1.5 mg/mL, 0.15 mg/mL and 0.015 mg/mL and subsequently incubated for 24 h at 37 °C. After washing with sterile PBS, the biofilm cells were collected by centrifugation (5000× *g* for 10 min) and stored at −80 °C. Total RNA was isolated and purified by means of RiboPure™ Bacteria Kit (ThermoFisher SCIENTIFIC, Waltham, MA, USA) according to the manufacturer′s instructions. To remove the residual DNA contaminating the purified RNA, the samples were treated with RNase-free DNase I. Integrity of RNA was verified by agarose gel electrophoresis. Its purity and concentration were determined by ND-8000 spectrophotometer (ThermoFisher SCIENTIFIC, Waltham, MA, USA). The isolated RNA (1 µg) was transcribed to cDNA by reverse transcription using Maxima H Minus Reverse Transcriptase (ThermoFisher SCIENTIFIC, Waltham, MA, USA) and Random Hexamer primers. The degree of relative expression of *S. aureus* genes (biofilm-related genes) involved in formation of biofilm *fnbA*, *fnbB* (cell wall associated (CWA) genes of proteins, microbial surface components recognizing adhesive matrix molecules (MSCRAMMs) adhesin proteins), *srtA* (transpeptidase catalysing the covalent adhesion of CWA proteins to cell wall peptidoglycans), operon *icaADBC* (polysaccharide intercellular adhesins) and *agrA* (*quorum-sensing* gene) was determined by qRT-PCR. Amplification and detection of specific products was conducted in triplicate by a CFX 96 RT system (Bio-Rad, Hercules, CA, USA) and Luna^®^ Universal qPCR Master Mix (New England BioLabs, Ipswich, MA, USA) using thermal profiles and mixed components of reaction mixture according to the provided manual. Each reaction mixture contained 20 ng cDNA and primers used in the final concentration of 0.5 pM. Sequences of gene-specific primers and the reference gene encoding B gyrase subunit (*gyrB*), serving as an internal control for determination of gene expression level, are presented in Table 4. Some primers used in this study were designed by means of Primer3 software, according to selected sequences obtained from the GenBank database. A dissociation curve was constructed at the end of each reaction using the melting temperature from 50 °C to 95 °C and reading at 0.5 °C increments. For each qRT-PCR there were negative controls used without an added template. The relative mRNA levels of genes were determined by normalization of the value of Cq of the studied genes to the mean value of Cq of reference genes by means of analysis of the value 2^−ΔCq^. The qRT-PCR data were expressed as the fold change in the levels of expression of genes in *S. aureus* CCM4223 cells exposed to various concentrations of LPs in comparison with the level of expression of control cells cultivated without LPs (calibrators).

### 4.11. Statistical Analysis

The data were analyzed by GraphPad Prism 6.01 software (GraphPad Inc., San Diego, CA, USA). The results were evaluated by one-way analysis of variance (ANOVA) followed by a Dunnett test with *p* < 0.05 considered significant.

## 5. Conclusions

In conclusion, our study demonstrated that the antibiofilm effect of LPs (the mixture of surfactin, fengycin and iturin) from *B. amyloliquefaciens* 3/22 was the result of a decrease in the down-regulation of *fnbA*, *fnbB*, *sortaseA* and *icaADBC* operon genes expression in *S. aureus* CCM 4223 biofilm. Our results indicate the potential of LPs use in the prevention of biofilm-related infections. However, the detailed molecular mechanism of action of purified compounds LPs 3/22 must be investigated.

## Figures and Tables

**Table 1:** Results of BLASTn analysis of 16S rRNA and BS genes of *B. amyloliquefaciens* 3/22.

Gene	GenBank Sequence Identity/Accession Number
16S rRNA	*B. amyloliquefaciens* (100%)
	KF811045.1
*srfAA*	*B. subtilis* (97.95%)
	KC454625.1
*sfp*	*B. amyloliquefaciens* (99.56%)
	KX346253.1
*fenB*	NA

*fenD*	*B. amyloliquefaciens* (96.63%)
	KP453873.1
*ituD*	*B. amyloliquefaciens* (99.36%)
	FJ815155.1

NA—no fragment amplification was observed.

**Table 2:** The percentage of inhibition of biofilm formation by *S. aureus* CCM 4223.

Activity [%]	Concentration of LPs 3/22 [mg/mL]			
	15	1.5	0.15	0.015
Inhibition of biofilm formation	100.19 ± 4.14	84.46 ± 4.21	60.09 ± 2.06	38.84 ± 13.76

**Table 3:** The primers used to screen the genes responsible for BSs biosynthesis [30,77,78].

Biosurfactants	Gene	Sequence	PCR Product Size [bp]	Annealing Temperature [°C]
Surfactin	*sfp*	F-5′ATGAAGATTTACGGAATTTA 3′ R-5′TTATAAAAGCTCTTCGTACG 3′	675	50
	*srfAA*	F-5′TCGGGACAGGAAGACATCAT 3′ R-5′CCACTCAAACGGATAATCCTGA 3′	201	60
Fengycin	*fenB*	F-5′CCTGGAGAAAGAATATACCGTACCY 3′ R-5′GCTGGTTCAGTT KGATCACAT 3′	670	57
	*fenD*	F-5′GGCCCGTTCTCTAAATCCAT 3′ F-5′GTCATGCTGACGAGAGCAAA 3′	269	60
Iturin A	*ituD*	F-5′ TTGAAYGTCAGYGCSCCTTT 3′ R-5′ TGCGMAAATAATGGSGTCGT 3′	482	57

**Table 4:** Primers used in the study of gene expression in *S. aureus* CCM4223.

Gene	Primer	Primer Sequence (5′-3′)	Reference
*fnbA*	fnbA-F	GAAGTGGCACAGCCAAGAAC	This study
	fnbA-R	ACGTTGACCAGCATGTGG	
*fnbB*	fnbB-F	CAATGATCCTATCATTGAGAAGAGTG	This study
	fnbB-R	CCTTCTACACCTTCAACAGCTGTA	
*srtA*	srtA-F	GTGGTACTTATCCTAGTGGCAGC	This study
	srtA-R	GCCTGCCACTTTCGATTTATC	
*icaA*	icaA-F	CTTGCTGGCGCAGTCAATAC	[82]
	icaA-R	GTAGCCAACGTCGACAACTG	
*icaD*	icaD-F	ACCCAACGCTAAAATCATCG	[83]
	icaD-R	GCGAAAATGCCCATAGTTTC	
*icaB*	icaB-F	ATACCGGCGACTGGGTTTAT	[83]
	icaB-R	ATGCAAATCGTGGGTATGTGT	
*icaC*	icaC-F	CTTGGGTATTTGCACGCATT	[83]
	icaC-R	GCAATATCATGCCGACACCT	
*agrA*	agrA-F	TCGTAAGCATGACCCAGTTG	This study
	agrA-R	AAATCCATCGCTGCAACTTT	
*gyrB*	gyrB-F	CCAGGTAAATTAGCCGATTGC	[84]
	gyrB-R	ATCGCCTGCGTTCTAGAGTC	

## Data Availability

The data presented in this study are available in this study.
